# Lymphoid-like Suppressive Microglia in Alzheimer’s Disease: A New Neuroimmune Regulatory Axis?

**DOI:** 10.3390/cells15131151

**Published:** 2026-06-24

**Authors:** James Chmiel, Wiktor Gawełczyk, Julia Soczyńska, Jerzy Leszek

**Affiliations:** 1 Faculty of Physical Culture and Health, Institute of Physical Culture Sciences, University of Szczecin, Al. Piastów 40B Block 6, 71-065 Szczecin, Poland; 2 Student Scientific Group of Psychiatry, Wroclaw Medical University, Wybrzeże L. Pasteura 10, 50-367 Wroclaw, Poland; wiktor.gawelczyk@student.umw.edu.pl (W.G.); julia.niznik@student.umw.edu.pl (J.S.); 3 Department of Psychiatry, Wroclaw Medical University, 50-367 Wroclaw, Poland

**Keywords:** Alzheimer’s disease, microglia, lymphoid-like microglia, suppressive microglia, neuroinflammation, PU.1, SPI1, CD28, disease-associated microglia, amyloid plaques, plaque-associated microglia, microglial heterogeneity, TREM2, APOE, complement, neuroimmune regulation, microglial reprogramming, single-cell transcriptomics, spatial transcriptomics, immunoregulatory microglia

## Abstract

**Highlights:**

- First bullet.PU.1-low CD28+ microglia may define a suppressive state in AD.
- The review links SPI1 genetics with plaque-niche microglial regulation.
- PU.1 dosage is framed as a context-dependent microglial state switch.
- CD28/B7-like signaling is proposed to restrain plaque inflammation.
- Human validation is needed before therapeutic translation is considered.

**Abstract:**

Microglia are central regulators of Alzheimer’s disease pathogenesis, but their roles cannot be reduced to a simple protective-versus-harmful dichotomy. Genetic, single-cell, and spatial studies have shown that Alzheimer ’s-associated microglia occupy diverse disease-linked states shaped by amyloid plaques, tau pathology, lipid stress, complement activation, astrocyte signaling, aging, and immune genetic risk. Among the regulatory nodes controlling these states, SPI1, which encodes the myeloid transcription factor PU.1, has emerged as a key determinant of microglial identity and disease responsiveness. Human genetic studies suggest that reduced SPI1 expression may be protective, whereas experimental data indicate that excessive PU.1 suppression can impair essential microglial functions. This review examines the emerging concept that partial, plaque-associated reduction in PU.1 may enable a distinct lymphoid-like immunoregulatory microglial program marked by CD28 expression. Recent evidence suggests that PU.1-low CD28-positive microglia may restrain neuroinflammation and amyloid pathology, raising the possibility that Alzheimer’s plaques induce not only inflammatory and phagocytic microglial responses, but also endogenous suppressive programs that limit tissue damage. We discuss this proposed PU.1/CD28 regulatory axis in relation to disease-associated microglia, TREM2–APOE signaling, complement-mediated synapse loss, antigen-presentation pathways, plaque-niche biology, and therapeutic microglial reprogramming. We also highlight major unresolved questions, including whether PU.1-low CD28-positive microglia are present and functional in human Alzheimer’s disease, whether they are specific to amyloid-rich niches or extend to tau and mixed pathologies, and how such states could be safely manipulated without disrupting essential immune surveillance. We propose that lymphoid-like suppressive microglia represent a promising but still unproven framework for understanding protective neuroimmune regulation in Alzheimer’s disease and for developing state-specific microglial therapies.

## 1. Introduction

Alzheimer’s disease has traditionally been framed around the accumulation of amyloid-β plaques, tau pathology, synaptic failure, and progressive cognitive decline, but the last decade has made it increasingly difficult to discuss these processes without placing microglia at the center of the disease landscape [1,2,3]. Microglia are the resident immune cells of the central nervous system [4], historically described as “resting” in the healthy brain and “activated” in disease [5]. This older binary language is now considered too limited. Even in homeostasis, microglia continuously survey the parenchyma [6], support neuronal integrity [7], contribute to synaptic remodeling [8], release trophic factors [8], and clear debris [9]; in disease, these same capacities can become either protective or damaging depending on context, timing, and molecular state [10,11,12]. Early neuroinflammatory models emphasized chronic activation as a driver of neuronal injury, but more recent work suggests that microglial responses in Alzheimer’s disease are not uniformly harmful and cannot be reduced to a simple activated-versus-resting distinction [13,14].

This shift has been driven partly by genetics. Large human genetic studies have repeatedly implicated immune and microglia-enriched genes in Alzheimer’s risk, including TREM2, PLCG2, and ABI3. The study by Sims et al. identified rare coding variants in these genes and argued that microglia-mediated innate immunity contributes directly to Alzheimer’s disease biology, rather than merely representing a secondary inflammatory reaction to plaques and tangles [15]. Around the same period, mechanistic studies of the TREM2–APOE pathway showed that microglial phenotypes in neurodegeneration are actively regulated transcriptional programs, not passive responses to tissue damage [16]. Together, these findings helped reframe microglia as genetically and functionally embedded in disease progression.

Single-cell and single-nucleus transcriptomic studies further transformed this field by revealing that Alzheimer ’s-associated microglia are heterogeneous. In 2017, Keren-Shaul et al. described disease-associated microglia as a distinct microglial type associated with neurodegenerative pathology, helping establish the idea that microglia can enter reproducible disease-linked transcriptional states [17]. Subsequent human studies extended this concept, showing that postmortem human microglia include Alzheimer ’s-associated subsets and that microglial signatures differ across individuals, disease states, and technical platforms [18]. These studies made it clear that microglia in Alzheimer’s disease should be understood as a spectrum of states shaped by plaque proximity, age, genetic background, metabolic stress, lipid handling, interferon signaling, complement activity, phagocytosis, and interactions with neurons and astrocytes.

The central unresolved issue is that the same broad category of “microglial activation” can describe processes with opposite biological consequences. Microglia may help contain amyloid plaques, phagocytose pathological material, support synapses, and limit inflammatory spread; yet they may also release pro-inflammatory mediators, promote complement-associated synapse loss, propagate neurotoxic signaling, and contribute to chronic tissue damage. Reviews and experimental studies now increasingly describe Alzheimer’s neuroinflammation as a dynamic balance between protective and harmful microglial programs, with disease stage and local tissue environment determining whether immune activation is beneficial, insufficient, maladaptive, or toxic [13,19]. This more nuanced view is important because it changes the therapeutic question: the aim may not be to suppress microglia broadly, but to identify and enhance protective microglial functions while limiting inflammatory or degenerative ones.

Within this context, the recent identification of PU.1-low, CD28-expressing microglia is especially interesting. Ayata et al. reported that microglial contact with amyloid plaques can downregulate the transcription factor PU.1, and that lower PU.1 expression is linked to a lymphoid-like immunoregulatory program marked by receptors such as CD28. Their study suggests that a small population of plaque-associated PU.1-low CD28-positive microglia may act as suppressive or neuroprotective microglia, reducing neuroinflammation and amyloid pathology in experimental models [20]. This finding builds directly on the broader evolution of the field: from microglia as generic inflammatory cells, to microglia as genetically implicated actors in Alzheimer’s disease, to microglia as heterogeneous cell states with separable protective and harmful functions.

The rationale for reviewing lymphoid-like suppressive microglia therefore rests on a clear gap. Alzheimer’s disease already has extensive reviews on neuroinflammation, TREM2, disease-associated microglia, and microglial activation states. However, the possibility that microglia may adopt an immune-regulatory program resembling lymphoid co-stimulatory or co-inhibitory signaling is newer and more specific. It raises questions that are not fully answered by the existing microglia literature: whether CD28-positive microglia represent a stable state or transient plaque-proximal response; whether PU.1 dosage acts as a therapeutic switch; whether similar cells exist in human Alzheimer’s disease across disease stages; and whether lymphoid-like signaling in microglia can be manipulated without impairing essential innate immune functions. This emerging area deserves a focused review because it links Alzheimer’s genetics, single-cell microglial state biology, immune-checkpoint signaling, plaque containment, and neuroprotective inflammation control into one coherent but still underdeveloped framework.

**Figure 1:**
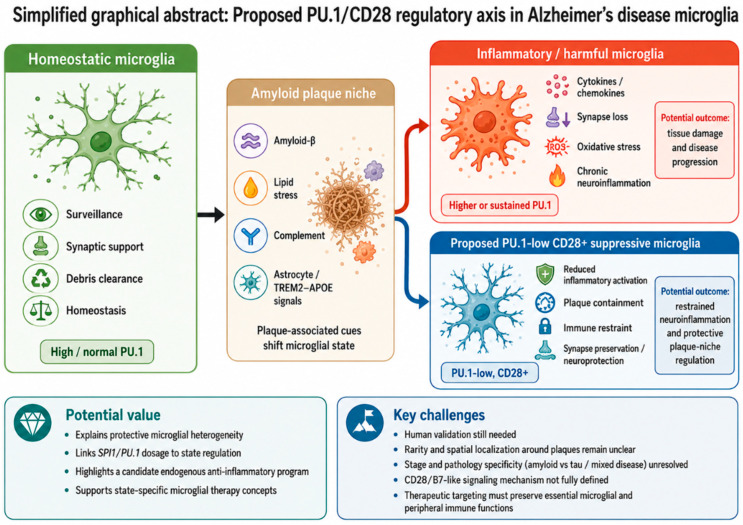
This schematic summarizes the central concept of the review. Amyloid plaque-associated cues may shift microglia toward either inflammatory/harmful states characterized by sustained PU.1 activity, cytokine production, oxidative stress, synapse loss, and chronic neuroinflammation, or toward a proposed PU.1-low CD28-positive suppressive state associated with immune restraint, plaque containment, reduced inflammatory activation, and possible synapse preservation. The lower panels highlight the potential value of this framework and the main unresolved challenges, including human validation, spatial localization, disease-stage specificity, CD28/B7-like signaling mechanisms, and therapeutic safety.

**Figure 2:**
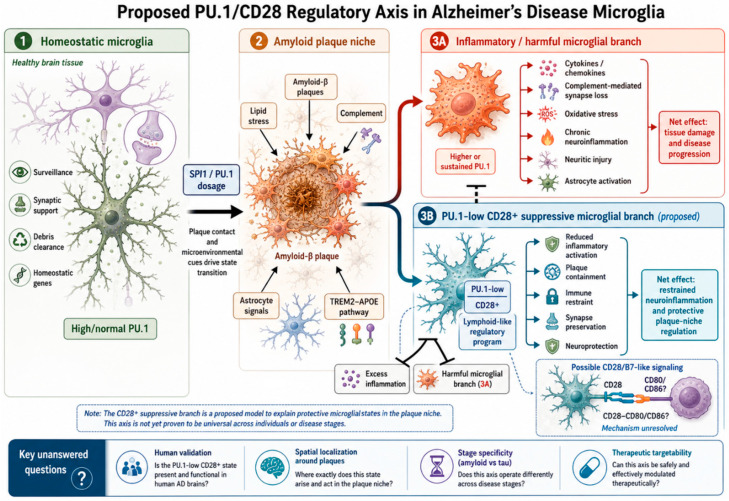
Proposed PU.1/CD28 regulatory axis in Alzheimer’s disease microglia. In healthy brain tissue, homeostatic microglia maintain surveillance, synaptic support, debris clearance, and homeostatic gene expression under high or normal PU.1 activity. In Alzheimer’s disease, amyloid-β plaques and associated plaque-niche cues, including lipid stress, complement activation, astrocyte-derived signals, and TREM2–APOE-related pathways, may drive microglial state transitions influenced by SPI1/PU.1 dosage. One branch of plaque-associated activation may produce inflammatory or harmful microglia characterized by sustained or elevated PU.1 activity, cytokine and chemokine release, oxidative stress, complement-mediated synapse loss, chronic neuroinflammation, neuritic injury, and astrocyte activation, with a net effect of tissue damage and disease progression. A second, still-proposed branch involves PU.1-low CD28-positive microglia with a lymphoid-like regulatory program that may reduce inflammatory activation, support plaque containment, preserve synapses, and promote neuroprotection. Possible CD28/B7-like signaling involving CD80/CD86 remains mechanistically unresolved. Key unanswered questions include whether this state is present and functional in human Alzheimer’s disease, where it localizes around plaques, whether it differs across amyloid- and tau-dominant disease stages, and whether it can be safely targeted therapeutically.

To provide readers with a concise overview of this concept, Figure 1 presents a simplified graphical abstract summarizing the two main plaque-associated microglial trajectories discussed in this review: an inflammatory/harmful branch and a proposed PU.1-low CD28-positive suppressive branch. The figure also highlights the potential value of this framework, including its relevance to protective microglial heterogeneity and state-specific therapeutic modulation, as well as the major unresolved challenges, such as human validation, spatial localization, disease-stage specificity, and the uncertain mechanisms of CD28/B7-like signaling. Figure 2 then presents a more detailed mechanistic model of the proposed PU.1/CD28 regulatory axis within the amyloid plaque niche.

## 2. Known Protective and Harmful Microglial States

The current understanding of microglia in Alzheimer’s disease is shaped by an apparent paradox: the same cell type can restrict pathology, clear toxic material, and support tissue repair, while also amplifying inflammation, promoting synapse loss, and contributing to neurodegeneration. This duality is not simply a matter of microglia being “good” early and “bad” late, although disease stage is important. Rather, microglia appear to occupy a range of molecular and functional states whose effects depend on local cues such as amyloid plaques, tau pathology, neuronal injury, lipid stress, complement activation, vascular dysfunction, aging, and genetic background. For this reason, the field has gradually moved away from broad terms such as “activated microglia” and toward more precise state-based descriptions, including disease-associated microglia, microglial neurodegenerative phenotype, interferon-responsive microglia, lipid-associated microglia, plaque-associated microglia, and inflammatory or senescent-like states.

A major turning point came from single-cell transcriptomic work in mouse models of Alzheimer’s disease. Keren-Shaul et al. described a population called disease-associated microglia, or DAM, which appeared around neurodegenerative pathology and showed a transcriptional profile distinct from homeostatic microglia. DAM were characterized by reduced expression of homeostatic genes and increased expression of genes related to phagocytosis, lipid metabolism, lysosomal activity, and Alzheimer’s risk pathways. Importantly, the authors proposed that DAM activation occurred in stages, with an initial TREM2-independent phase followed by a TREM2-dependent program. This was significant because it suggested that microglia do not merely become inflamed in a nonspecific way; they can enter organized disease-response programs that may help contain pathology by phagocytosing amyloid-β and responding to damaged tissue [17].

A related concept emerged from Krasemann et al., who described a microglial neurodegenerative phenotype, often abbreviated as MGnD. In this model, the transition from homeostatic microglia toward a neurodegeneration-associated state was regulated by the TREM2–APOE pathway. The study showed that microglia in neurodegenerative settings downregulated homeostatic genes and upregulated genes associated with lipid handling, phagocytosis, and inflammatory responses. The authors emphasized that this switch was not unique to Alzheimer’s disease but reflected a broader microglial response to neurodegeneration. However, the interpretation of MGnD was more ambivalent than a purely protective DAM model: while some components of the response may support clearance of debris and pathological aggregates, the same program was also linked to loss of homeostatic or tolerogenic functions [16].

These studies created a productive tension in the literature. On one hand, disease-associated microglia can be viewed as protective because they cluster around plaques, increase phagocytic and lysosomal programs, and may physically compact amyloid deposits, thereby limiting plaque-associated neuritic damage. This protective interpretation is consistent with the observation that TREM2 loss-of-function variants increase Alzheimer’s disease risk and that impaired TREM2 signaling can reduce the ability of microglia to respond effectively to amyloid pathology. On the other hand, the same disease-associated programs may become maladaptive if they persist chronically, fail to clear pathology, lose homeostatic support functions, or produce inflammatory mediators that damage nearby neurons and synapses. Thus, DAM and MGnD are best understood not as inherently beneficial or harmful categories, but as context-dependent responses whose net effect depends on disease stage, brain region, and the balance between phagocytic containment and inflammatory injury.

Human single-cell and single-nucleus studies complicated this picture further. Olah et al. used single-cell RNA sequencing of human microglia and identified subsets enriched for disease-related genes and Alzheimer ’s-associated signatures, supporting the idea that microglial heterogeneity observed in mice has relevance to human disease. However, human microglial states do not always map neatly onto mouse DAM or MGnD categories. Human postmortem tissue is affected by age, agonal state, disease duration, brain region, postmortem interval, and technical differences between single-cell and single-nucleus approaches. As a result, human Alzheimer’s microglia often show overlapping but non-identical signatures involving lipid metabolism, immune activation, stress responses, antigen presentation, interferon signaling, and phagocytic pathways. This has made the field more cautious about treating any single-named microglial state as a universal Alzheimer’s phenotype [18].

Later transcriptomic studies reinforced this caution by showing that Alzheimer ’s-associated microglial states vary with amyloid, tau, and individual-level disease context. Zhou et al. compared human Alzheimer’s disease and 5XFAD mouse tissue using single-nucleus RNA sequencing, linking disease-associated microglial changes to both AD pathology and TREM2-related biology [21]. More recent human work has described microglial state dynamics across large postmortem cohorts, suggesting that microglial populations shift along trajectories rather than falling into a few fixed categories [22]. This trajectory-based view is important for a review of suppressive or lymphoid-like microglia because it leaves room for small, transient, spatially restricted, or disease-stage-specific populations that may have been missed or collapsed into broader inflammatory clusters in earlier analyses.

The harmful side of microglial biology is perhaps clearest in studies of synapse loss. Synaptic dysfunction and synapse loss correlate strongly with cognitive decline in Alzheimer’s disease, and microglia have been implicated in aberrant synaptic pruning through complement pathways. Hong et al. showed that complement and microglia mediate early synapse loss in mouse models of Alzheimer’s disease, with C1q and downstream complement signaling tagging synapses for removal before extensive plaque deposition [23]. This work was influential because it connected a normal developmental function of microglia—synaptic refinement—to a pathological process in neurodegeneration. In this setting, microglial phagocytosis is not simply beneficial clearance; it can become misdirected toward vulnerable synapses, contributing directly to circuit failure.

The complementary literature also illustrates why classifying microglia as either protective or harmful is difficult. Complement-dependent synapse elimination may be damaging when it removes functional synapses, but complement proteins can also participate in debris clearance, immune surveillance, and tissue remodeling. More recent work has expanded this framework by showing that astrocytes, microglia, and complement pathways interact in complex ways during amyloid and tau pathology. For example, Dejanovic et al. reported that C1q-dependent mechanisms can involve astrocytic synapse elimination and can compensate for altered microglial phagocytic function in an Alzheimer’s model combining amyloid and tau pathologies. This suggests that harmful microglial states cannot be understood in isolation; they are embedded within a glial network in which astrocytes, neurons, complement proteins, and peripheral immune signals shape the final outcome [24].

Inflammatory microglial states represent another potentially harmful axis. In Alzheimer’s disease, microglia can produce cytokines, chemokines, reactive oxygen species, inflammasome-associated mediators, and other signals that may worsen neuronal stress. Chronic inflammatory activation may also impair microglial phagocytosis, alter metabolism, reduce homeostatic surveillance, and contribute to a feed-forward cycle in which amyloid, tau, and cellular injury sustain immune activation. However, inflammation is not universally detrimental. Some inflammatory pathways may help recruit microglia to pathology, promote aggregate clearance, or support tissue repair. The key problem is therefore not inflammation itself, but the failure to resolve inflammation or to coordinate it with effective clearance and repair. This is why broad anti-inflammatory approaches have generally been less successful than expected, whereas targeted modulation of specific microglial pathways remains an active therapeutic goal.

Protective microglial states are often defined by their ability to surround plaques, compact amyloid, clear debris, maintain barriers around pathology, regulate lipid stress, and prevent inflammatory spread. Harmful states are often defined by excessive cytokine production, complement-mediated synapse engulfment, impaired homeostatic support, oxidative stress, antigen-presenting or interferon-driven activation, and contribution to neuronal dysfunction. Yet many Alzheimer ’s-associated microglial programs contain both sets of features. A plaque-associated microglial cell may express phagocytic and lysosomal genes that help contain amyloid, while also expressing inflammatory mediators that could damage synapses. A TREM2-dependent response may be necessary for effective plaque engagement, but insufficient or prolonged activation may contribute to chronic neuroimmune dysfunction. A complement-engaged microglial program may remove damaged synapses and debris, but may also eliminate synapses that are stressed yet still functional.

Recent work on MEF2C further supports the idea that Alzheimer ’s-associated microglial states are governed by transcription-factor dosage rather than by a simple activated-versus-resting switch. Podlesny-Drabiniok et al. identified MEF2C as a candidate Alzheimer’s disease risk gene and a regulator of disease- and lipid-associated macrophage/microglial programs. In human iPSC-derived microglia and macrophage models, total or partial MEF2C reduction was sufficient to induce a DLAM-like state characterized by lysosomal, phagocytic, lipid-handling, and cholesterol-efflux pathways. Importantly, the authors linked MEF2C-regulated regulatory elements to candidate Alzheimer’s disease risk genes and showed that, in a human triculture model containing Alzheimer’s disease neurons and astrocytes, MEF2C-deficient microglia were associated with an increased DLAM population and a reduced Aβ42/40 ratio [25].

This finding is relevant to the present review because it places PU.1-low CD28-positive microglia within a broader principle of microglial state regulation. Disease-associated, lipid-associated, and potentially suppressive microglial programs may emerge when key myeloid transcription factors are shifted quantitatively rather than completely removed. However, MEF2C also illustrates the need for caution. Neuronal MEF2C supports synaptic and cognitive resilience, whereas chronic or developmental loss of MEF2C in microglia may have detrimental consequences. Thus, as with PU.1, the biological effect of MEF2C reduction depends on cell type, timing, magnitude, and disease context. These observations strengthen the view that protective Alzheimer ’s-associated microglial responses may require precise state tuning rather than broad activation or suppression.

This ambiguity is precisely why newly described microglial populations such as PU.1-low, CD28-positive lymphoid-like microglia are important. Existing categories such as DAM and MGnD have already shown that microglia can enter disease-linked states, but they do not fully explain how protective immune regulation is achieved within the Alzheimer’s brain. The field has many models of microglial activation, phagocytosis, and inflammatory injury, but fewer frameworks for microglial suppression, immune restraint, and active resolution of neuroinflammation. A lymphoid-like suppressive microglial state would therefore add a missing layer to the current taxonomy: not simply homeostatic microglia, inflammatory microglia, or plaque-clearing microglia, but microglia that may regulate the intensity and consequences of neuroimmune activation. Section 3 can build from this point by asking how SPI1/PU.1 dosage influences the transition between homeostatic, disease-associated, and potentially suppressive microglial states.

## 3. PU.1/SPI1 in Alzheimer’s Disease

PU.1, encoded by SPI1, is one of the most important transcription factors for understanding why Alzheimer’s disease is now so closely linked to microglial biology. PU.1 belongs to the ETS family of transcription factors and is central to myeloid lineage specification, including the development and functional identity of macrophages and microglia. In the brain, PU.1 helps organize the enhancer landscape that allows microglia to maintain their identity, respond to environmental signals, and activate disease-relevant gene programs. This means that SPI1 is not simply another Alzheimer ’s-associated gene; it is a regulatory node capable of shaping many downstream immune, phagocytic, lipid-handling, and inflammatory pathways at once. Reviews of microglial transcriptional regulation have therefore placed PU.1 alongside factors such as IRF8, SALL1, SMAD proteins, and other enhancer-associated regulators that define microglial identity and reactivity [26,27].

PU.1 should also be interpreted in relation to interacting enhancer-associated transcription factors rather than as an isolated regulator. Saeki et al. recently showed that IRF8 configures the postnatal microglial enhancer landscape through stepwise binding to enhancer regions together with SALL1 and PU.1. In their model, IRF8 binding preceded and supported chromatin accessibility, DNA methylation patterns, and microglia-specific transcriptional maturation. Constitutive or postnatal Irf8 deletion caused a loss of microglial identity genes and a gain of disease-associated microglia-like and interferon-related genes, indicating that disruption of one core transcription factor can redirect microglia toward disease-like states [28].

This study is important for the PU.1/CD28 framework because it shows that microglial disease states can arise from altered enhancer logic, not only from direct plaque stimulation. IRF8 interacts with PU.1 at composite ETS/IRF regulatory elements, and changes in IRF8-dependent chromatin accessibility can expose alternative transcriptional programs, including DAM-like and lysosomal/CLEAR-related genes. In the 5xFAD model, Irf8 deletion also reduced microglial interaction with amyloid plaques and was associated with reduced plaque size and less neuronal loss. These findings should not be interpreted as evidence that suppressing microglial identity is universally beneficial. Rather, they reinforce the broader concept that Alzheimer’s pathology is shaped by the balance between homeostatic identity, plaque-associated activation, lipid/lysosomal clearance, and inflammatory programs. In this sense, PU.1-low CD28-positive microglia may represent one specific outcome within a wider transcription-factor network that includes PU.1, IRF8, SALL1, MEF2C, and other state-regulating factors.

The importance of SPI1 in Alzheimer’s disease first became especially clear through human genetic and functional genomic studies. A common protective haplotype associated with lower SPI1 expression was reported to delay Alzheimer’s disease onset, suggesting that reduced PU.1 abundance in myeloid cells may be beneficial under some disease-relevant conditions. The key implication was that Alzheimer’s risk could be influenced not only by coding changes in immune genes, but also by regulatory variants that alter how strongly a myeloid transcription factor is expressed. This was an important conceptual shift because it linked inherited disease risk to microglial transcriptional dosage rather than to a single downstream effector pathway [12,29].

Subsequent work strengthened this interpretation by showing that SPI1/PU.1 sits near the center of Alzheimer’s-related microglial regulatory networks. In human microglial regulome studies, transcription factor network analyses nominated SPI1 as a key regulator of microglial gene expression and Alzheimer’s disease risk, helping connect noncoding genetic risk loci to cell-type-specific gene regulation [30]. This is significant because many Alzheimer’s risk variants lie outside protein-coding regions and are enriched in immune or myeloid regulatory elements. SPI1 therefore provides a plausible bridge between genome-wide association signals and altered microglial behavior in disease. Instead of asking only which risk genes are expressed in microglia, the field has increasingly asked how Alzheimer’s risk variants reshape the regulatory architecture through which microglia interpret amyloid, tau, aging, and tissue injury.

Experimental modulation of PU.1 has also shown that its expression level can alter microglial phenotype. Pimenova et al. examined Alzheimer’s-associated PU.1 expression levels in microglial models and reported that higher SPI1 expression is associated with increased Alzheimer’s risk, whereas lower expression is associated with protection. In their experimental system, reducing PU.1 expression shifted the microglial transcriptome in a way that overlapped with disease-associated microglial responses to amyloid plaques [31]. This finding is particularly relevant to the present review topic because it suggests that PU.1 dosage may influence whether microglia remain homeostatic, become classically inflammatory, or enter an Alzheimer’s-associated response state. The relationship is unlikely to be linear or universally protective, but the basic point is clear: modest changes in PU.1 abundance can have broad transcriptional consequences.

Other studies have complicated the idea that simply lowering PU.1 is always beneficial. Because PU.1 is required for microglial identity and viability, excessive reduction may impair essential functions. Earlier work in human microglia showed that PU.1 is critical for microglial viability and expression of characteristic microglial proteins, indicating that PU.1 cannot be viewed only as a disease-promoting factor [32]. Similarly, studies of Spi1 dosage in mice suggest that modest changes can reshape the microglial transcriptome, but the biological consequences depend on timing, magnitude, and disease context [33]. This creates a central tension for Alzheimer’s therapeutics: lowering PU.1 may reduce risk-associated or inflammatory programs, but too much suppression could compromise microglial survival, plaque engagement, phagocytosis, or other protective functions.

This tension became even more apparent in mechanistic studies that examined PU.1 in relation to amyloid pathology and microglial activation. PU.1 and IRF8 have been described as mutually reinforcing transcriptional regulators involved in microglial activation, with deletion of either factor impairing activation-related transcriptional programs [34]. More recent Alzheimer’s-focused work has suggested that PU.1 can directly influence TREM2 expression during amyloid-β-induced microglial activation [35]. Because TREM2 is central to plaque-associated microglial responses, lipid handling, and disease-associated microglial states, this places PU.1 upstream of one of the most intensively studied protective-versus-harmful microglial pathways in Alzheimer’s disease. PU.1 may therefore help determine not only whether microglia activate, but also what kind of activation state they adopt.

The 2024 work on SPI1-mediated transcriptome remodeling further illustrates why PU.1 remains difficult to classify as either a therapeutic target to inhibit or a factor to preserve. That study explicitly addressed whether SPI1 should be increased or decreased for therapeutic benefit, using both knockdown and overexpression approaches in Alzheimer’s-relevant models [36]. The fact that this question remains unresolved is important. Human genetics suggests that lower SPI1 expression can be protective, but microglial biology suggests that PU.1 is also required for essential myeloid identity and disease-response functions. Therefore, the most plausible therapeutic interpretation is not that PU.1 should be globally suppressed, but that PU.1 dosage may need to be tuned to favor protective microglial states while avoiding loss of core microglial function.

This dosage-based interpretation is directly relevant to the recent identification of PU.1-low, CD28-expressing microglia. Ayata et al. reported that microglial contact with amyloid plaques can reduce PU.1 abundance and induce a lymphoid-like gene-expression program, including CD28 expression, in a small microglial population. Their findings suggest that PU.1-low microglia may not simply represent weakened or dysfunctional cells; instead, they may acquire a suppressive, neuroprotective phenotype that limits inflammation and pathology [20]. This is a provocative extension of earlier SPI1 genetics. The protective SPI1 haplotype implied that lower PU.1 expression might reduce Alzheimer’s risk at the organismal level, while the newer study proposes a cellular mechanism by which local PU.1 reduction near plaques could generate a protective microglial state.

The key conceptual advance is that PU.1 may operate as a state-switching regulator rather than a simple on–off determinant of microglial activation. High or sustained PU.1 activity may support myeloid identity and certain inflammatory or phagocytic programs, whereas partial reduction may permit alternative transcriptional states, including disease-associated or lymphoid-like suppressive programs. This does not mean that all PU.1-low states are beneficial. A PU.1-low microglial cell could be protective, exhausted, developmentally unstable, or functionally impaired depending on context. However, the discovery of plaque-associated PU.1-low CD28-positive microglia gives the field a concrete reason to revisit older SPI1 findings through the lens of microglial heterogeneity.

For a review focused on lymphoid-like suppressive microglia, SPI1/PU.1 is therefore the logical bridge between Alzheimer’s genetics and microglial state biology. It links inherited risk, enhancer regulation, microglial identity, TREM2-associated activation, plaque responses, and immune-regulatory phenotypes. The open question is no longer simply whether SPI1 increases or decreases Alzheimer’s risk. The more interesting question is how different levels of PU.1 shape the balance between homeostatic maintenance, plaque containment, inflammatory injury, and suppressive neuroimmune regulation. Section 4 can build from this foundation by examining whether CD28 and other lymphoid-like signaling molecules in microglia represent a genuine immune-regulatory program or a transient transcriptional adaptation to the plaque microenvironment. To help orient the reader, Table 1 summarizes the key studies that shaped the conceptual basis of this review, from microglial genetic risk and disease-associated microglial states to SPI1/PU.1 dosage, CD28-positive suppressive microglia, and therapeutic microglial reprogramming.

## 4. CD28 and Lymphoid-like Signaling in Microglia

The lymphoid-like microglial concept should also be considered against the expanding literature on adaptive immune changes in Alzheimer’s disease. Recent human work combining CyTOF, single-cell RNA sequencing, T-cell secretome analysis, and antigen-presentation assays reported that CD4-positive T cells in preclinical Alzheimer’s disease show a type 2 helper profile and reactivity against Aβ-derived peptides, whereas MCI subjects show increased pro-inflammatory CD8-positive effector/TEMRA cells. This suggests that the adaptive immune compartment is not merely a late bystander but may change across disease stages. In mouse models, clonally expanded CD8-positive T cells have also been shown to interact dynamically with microglia: early CD8-positive T-cell activity can worsen amyloid pathology and constrain DAM-like microglial transition, whereas later T-cell states may become more tissue-resident or regulatory. These findings strengthen the rationale for treating CD28-positive microglia not as an isolated molecular curiosity, but as part of a broader stage-dependent neuroimmune circuit involving microglia, plaques, and adaptive immune cells [37,39].

The discovery of CD28-expressing PU.1-low microglia is striking because CD28 is not usually considered a defining microglial molecule. In classical immunology, CD28 is best known as a co-stimulatory receptor on T cells. Its engagement by the B7-family ligands CD80 and CD86 on antigen-presenting cells provides a second signal that supports T-cell activation, survival, proliferation, cytokine production, and differentiation. Without this co-stimulatory context, antigen recognition alone may lead to incomplete activation, tolerance, or anergy. Early studies in the 1990s established CD80 and CD86 as potent T-cell co-stimulators, while later reviews refined the view of CD28 as a receptor that does not simply “turn on” T cells, but tunes the quality, duration, and metabolic consequences of immune activation [40,41,42].

A useful way to frame CD28-positive microglia is to note that CD28 expression outside classical T cells is unusual but not without precedent. In peripheral myeloid biology, CD28 has been reported in anti-inflammatory macrophages, where it was proposed as a marker of suppressive or regulatory macrophage polarization rather than conventional lymphocyte activation [43]. This precedent does not prove that microglial CD28 has the same function, but it makes the Ayata et al. finding more plausible: CD28 expression in a myeloid cell may mark an immunoregulatory state rather than a lineage error or sequencing artifact. The key implication is that CD28 should be interpreted as part of a broader regulatory module whose function must be defined in microglia, not inferred directly from T-cell co-stimulation.

For this reason, CD28 expression in microglia raises a conceptual problem. Microglia are myeloid cells, not lymphocytes, and they have usually been discussed in Alzheimer’s disease through frameworks such as phagocytosis, cytokine release, complement activation, lipid handling, inflammasome signaling, and plaque containment. They can express molecules associated with antigen presentation, including MHC class II–related genes and co-stimulatory ligands such as CD80 or CD86, especially under inflammatory conditions. However, this is different from microglia expressing a receptor such as CD28, which is canonically positioned on the lymphocyte side of the immune synapse. The recent finding therefore suggests that at least some plaque-associated microglia may borrow elements of lymphoid signaling logic rather than merely presenting antigen to lymphocytes [44,45,46].

Earlier work had already prepared the field for this idea by showing that microglia are capable of participating in adaptive immune-like interactions. In inflammatory and neurodegenerative settings, microglia can upregulate antigen-presentation machinery, respond to interferon-γ and other lymphocyte-derived cytokines, and influence T-cell behavior within or near the central nervous system. Reviews of microglia–lymphocyte communication have emphasized that microglia may act as local regulators of lymphocyte activation, particularly when blood–brain barrier dysfunction or chronic neuroinflammation allows greater immune-cell trafficking into the CNS. In this framework, molecules such as MHC-II, CD80, CD86, CD40, and related co-stimulatory or co-inhibitory pathways become relevant because they determine whether immune recognition leads to activation, restraint, or tolerance [44,45].

Alzheimer’s disease adds another layer to this problem because the diseased brain contains persistent protein aggregates, neuronal damage, lipid debris, complement activation, and age-related immune remodeling. Single-cell and single-nucleus studies have repeatedly identified Alzheimer ’s-associated microglial states enriched for immune activation, antigen-presentation genes, lipid-processing pathways, and inflammatory mediators. These states do not map cleanly onto a simple innate-versus-adaptive immune division. Instead, microglia in Alzheimer’s disease appear to acquire hybrid programs: they remain tissue-resident myeloid cells, but they can express genes normally associated with professional antigen presentation, immune communication, and inflammatory coordination. Reviews of single-cell microglial biology in Alzheimer’s disease have therefore highlighted MHC-II-positive and antigen-presentation-associated microglial populations as part of the broader disease-state spectrum [18,46,47].

The lymphoid-like CD28 signal described by Ayata et al. is more specific than the earlier antigen-presentation literature. Their study reported that lowering PU.1 expression in microglia reduced amyloid disease severity in mice and was linked to expression of immunoregulatory lymphoid receptor proteins, particularly CD28. CD28 was expressed by a small subset of plaque-associated PU.1-low microglia, and microglia-specific CD28 deficiency promoted a broader inflammatory microglial state together with increased amyloid plaque burden. The authors therefore proposed that PU.1-low CD28-expressing microglia may function as suppressive microglia that mitigate Alzheimer’s progression by reducing neuroinflammation [20].

This interpretation is important because it reframes CD28 from a marker of lymphocyte activation into a possible microglial regulatory mechanism. In T cells, CD28 is usually discussed as an activating co-stimulatory receptor, but in microglia, the functional meaning may be different. The Ayata study suggests that CD28-positive microglia may restrain inflammatory activation rather than amplify it. This does not necessarily contradict classical immunology; rather, it shows that the same molecular module can have different consequences depending on the cell type, receptor context, ligand availability, transcriptional state, and tissue environment. In a plaque-associated microglial niche, CD28 may participate in a local feedback system that limits excessive inflammatory escalation while preserving protective plaque-associated functions [20,41].

The term “lymphoid-like” should therefore be used carefully. It does not imply that microglia become lymphocytes, nor that they perform T-cell receptor–dependent antigen recognition. Instead, it suggests that a subset of microglia can express receptors and regulatory programs more commonly associated with lymphoid immune control. This distinction matters for review framing. A review should avoid overstating the phenomenon as a complete lineage conversion. The more defensible claim is that Alzheimer’s pathology may induce microglial states that incorporate selected lymphoid co-stimulatory or co-inhibitory signaling components, possibly to regulate the intensity of neuroinflammation. That is a narrower and more biologically plausible hypothesis.

The plaque microenvironment may be central to this phenomenon. Amyloid plaques create a highly localized niche containing aggregated amyloid-β, dystrophic neurites, lipids, complement proteins, reactive astrocytes, cytokines, and metabolically stressed microglia. Many disease-associated microglial programs are spatially enriched around plaques, which suggests that microglial state transitions are not only disease-stage-dependent but also anatomically localized. The PU.1-low CD28-positive population appears to fit this spatial logic: it is not described as a bulk transformation of all microglia, but as a small plaque-associated subset. This may explain why such cells have not been the central focus of earlier Alzheimer’s microglia reviews, which often emphasized larger transcriptional clusters such as DAM, MGnD, interferon-response microglia, or MHC-II-positive microglia [20,46,47].

One unresolved question is whether CD28-positive microglia interact directly with canonical CD28 ligands. CD80 and CD86 are traditionally expressed by antigen-presenting cells and bind CD28 and CTLA-4 on T cells. In Alzheimer’s disease and aging, microglia and peripheral myeloid cells may express co-stimulatory ligands, but the ligand–receptor architecture within the plaque microenvironment remains incompletely defined. It is possible that CD28 on microglia responds to CD80/CD86 expressed by neighboring microglia, infiltrating immune cells, border-associated macrophages, or other antigen-presenting populations. It is also possible that CD28 has microglia-specific signaling partners or noncanonical functions that are not yet fully understood. These possibilities are precisely why this topic deserves review: the classical CD28 literature provides a mechanistic vocabulary, but Alzheimer’s microglia may use that vocabulary in a new cellular context [40,42,45].

Another important question is whether lymphoid-like signaling in microglia is protective only in amyloid pathology or also relevant to tau pathology, vascular injury, or mixed dementia. Many microglial responses differ between amyloid-dominant and tau-dominant contexts, and human Alzheimer’s disease rarely consists of amyloid pathology alone. The Ayata study is especially relevant to amyloid-associated plaque microenvironments, but a full review should ask whether PU.1-low CD28-positive microglia are present in human Alzheimer’s brain, whether they vary by Braak stage or plaque burden, and whether they appear in datasets enriched for tau pathology. Human microglia atlas studies and postmortem single-cell datasets may be particularly valuable here because they allow investigators to search for rare or spatially restricted immune-regulatory signatures across disease stages and brain regions [20,47].

This topic also connects to the broader therapeutic movement away from blanket anti-inflammatory strategies. If CD28-positive suppressive microglia are protective, then the goal would not be to suppress microglia globally, but to preserve or expand a regulatory state that restrains damaging inflammation while maintaining plaque containment and debris clearance. This would fit with the emerging view that Alzheimer’s immunotherapy may require state-specific microglial modulation rather than simple activation or inhibition. However, the therapeutic implications are delicate. CD28 and B7-family pathways are deeply involved in peripheral immune regulation, so systemic manipulation could affect T-cell responses, autoimmunity, infection risk, or immunosurveillance. A microglia-targeted or CNS-restricted approach would therefore be more attractive than broad systemic CD28 pathway modulation.

Overall, CD28 and lymphoid-like signaling in microglia represent a new way of thinking about Alzheimer’s neuroinflammation. Earlier models emphasized microglia as innate immune cells that clear plaques or produce inflammatory mediators. Later single-cell studies showed that microglia occupy multiple disease-associated states, including antigen-presenting and inflammatory populations. The newer CD28-positive PU.1-low microglial finding adds a further possibility: some microglia may adopt an active immune-regulatory program that suppresses excessive inflammation and protects the diseased brain. The value of reviewing this area lies in bringing together the literature that is usually treated separately—T-cell co-stimulation, microglial antigen presentation, Alzheimer’s single-cell biology, PU.1 dosage, plaque-associated microglial states, and neuroprotective immune regulation—into a single framework.

## 5. Therapeutic Implications

The therapeutic significance of lymphoid-like suppressive microglia lies in the possibility that Alzheimer’s disease treatment may need to move beyond the older goal of broadly suppressing neuroinflammation. For many years, inflammation in Alzheimer’s disease was interpreted mainly as a damaging accompaniment of amyloid plaques and neuronal injury. This view encouraged interest in anti-inflammatory strategies such as non-steroidal anti-inflammatory drugs, because epidemiological studies and experimental models suggested that inflammatory mediators and activated microglia were associated with Alzheimer’s pathology. Earlier reviews summarized this rationale by noting that activated microglia and inflammatory pathways are prominent around Alzheimer’s lesions, and that NSAIDs had shown protective associations in some observational studies and animal models. However, the clinical translation of broad anti-inflammatory approaches has been disappointing, partly because inflammation in Alzheimer’s disease is not a single harmful process that can simply be turned off [48,49].

This history is important because it explains why a PU.1-low, CD28-positive microglial state is therapeutically interesting. If some microglial responses are protective while others are damaging, then global microglial inhibition may remove beneficial functions along with harmful ones. Microglia can compact plaques, clear amyloid-β, remove debris, regulate lipid stress, support repair, and coordinate local tissue responses, even while they can also produce cytokines, engage complement-mediated synapse loss, and contribute to chronic inflammatory injury. Recent reviews of neuroinflammation therefore emphasize that the challenge is not simply to reduce inflammation, but to modulate it with enough precision to preserve protective microglial activity while preventing maladaptive activation [50,51].

In this context, the central therapeutic implication of PU.1-low CD28-positive microglia is state tuning. Ayata et al. reported that microglial contact with amyloid plaques can reduce PU.1 and promote a lymphoid-like program, including CD28 expression, in a small plaque-associated microglial subset. They further suggested that these PU.1-low CD28-expressing cells may act as suppressive microglia that reduce neuroinflammation and mitigate Alzheimer’s progression [20]. The attractive therapeutic idea is therefore not to silence microglia, but to increase the probability that disease-exposed microglia adopt a regulatory, plaque-protective, inflammation-limiting state.

The therapeutic relevance of PU.1 dosage is further supported by pharmacological work targeting PU.1-dependent inflammatory transcription. Ralvenius et al. identified A11, a small molecule that modulates PU.1-regulated gene expression by recruiting a repressive MECP2/HDAC1-containing complex to PU.1 motifs rather than simply eliminating PU.1 expression. In human iPSC-derived microglia-like cells and mouse models, A11 reduced inflammatory PU.1-target gene programs, including genes involved in innate immune and cytokine responses. In several transgenic mouse models representing different Alzheimer’s disease-related pathological features, including amyloid deposition, tauopathy, and neurodegeneration, A11 reduced neuroinflammation and neuropathology and improved cognitive performance [38].

This study is especially relevant because it supports a therapeutic model based on transcriptional restraint rather than global microglial suppression. A11 represents an example of how PU.1-dependent inflammatory activity might be dampened while avoiding complete disruption of PU.1 expression, which is essential for myeloid identity and hematopoiesis. The authors also reported no major effects on hematopoietic stem-cell proliferation or differentiation at tested concentrations and no major changes in circulating CD45-positive, CD4-positive, or CD8-positive cells after repeated treatment in mice. Nevertheless, these findings remain preclinical and should be interpreted cautiously [38]. The key implication for the present review is not that A11 is already a clinically validated Alzheimer’s therapy, but that PU.1-associated transcriptional programs are pharmacologically tractable and may be modulated in a more state-selective manner than older broad anti-inflammatory approaches.

This idea fits a broader movement in Alzheimer’s therapeutics toward microglial reprogramming. The best-developed example is TREM2, a microglial receptor involved in lipid sensing, survival, phagocytosis, metabolism, and plaque-associated microglial responses. TREM2 has attracted therapeutic interest because enhancing TREM2 signaling may strengthen the ability of microglia to respond to amyloid plaques and neuronal damage. Newer work continues to examine how TREM2 expression level and activation state influence microglial metabolism and phagocytic capacity, reinforcing the broader principle that microglial state, not merely plaque burden, is a plausible therapeutic target [52,53].

The PU.1/CD28 axis differs from TREM2; however, it points less toward enhancing plaque engagement alone and more toward enhancing immune restraint within the plaque niche. This distinction matters. TREM2-centered strategies often aim to improve microglial survival, clustering, lipid metabolism, and amyloid response. A CD28-positive suppressive-microglia strategy would instead ask whether a regulatory microglial subset can be expanded or stabilized so that plaque-associated microglia clear or contain pathology without escalating into damaging inflammation. In practical terms, this could mean identifying signals that induce the PU.1-low CD28-positive state, defining its ligand requirements, and testing whether the state can be promoted without impairing normal microglial surveillance or host defense.

PU.1 itself is an especially tempting but risky therapeutic node. Human genetic work has suggested that lower SPI1 expression can be associated with delayed Alzheimer’s disease onset, and experimental studies have shown that changing PU.1 levels can reshape microglial transcriptional programs. However, more recent in vivo work complicates a simple “lower PU.1 is better” interpretation. A 2024 study reported that Spi1 knockdown worsened multiple Alzheimer ’s-related pathological hallmarks, including amyloid aggregation, plaque accumulation, and gliosis, in mouse models [36]. This means that PU.1 cannot be treated as a conventional target for broad inhibition. It is a core myeloid transcription factor, and excessive or poorly timed suppression may damage the very microglial functions needed for amyloid response and tissue maintenance.

A more plausible therapeutic model is therefore partial, contextual, or cell-state-specific modulation of PU.1-regulated programs. The goal would not be to reduce SPI1 globally across all microglia, but to understand how plaque contact produces a local PU.1-low regulatory state and whether that state can be mimicked more selectively. This could involve targeting downstream effectors of the PU.1-low program rather than PU.1 itself. CD28 is the most obvious candidate, but other lymphoid-like co-stimulatory or co-inhibitory receptors identified in the same transcriptional program may eventually prove more druggable or safer. The review should therefore present PU.1 as a gateway into protective state biology, not as an immediate stand-alone drug target.

CD28 also presents both opportunity and danger. In peripheral immunology, CD28 is a central T-cell co-stimulatory receptor, and interventions affecting CD28 or its B7-family ligand network can have major consequences for immune activation, tolerance, autoimmunity, and infection risk. For Alzheimer’s disease, this raises a major translational problem: systemic manipulation of CD28 signaling could alter peripheral T-cell function in undesirable ways. Even if CD28-positive microglia are protective in the brain, a therapy that broadly stimulates or blocks CD28 pathways throughout the body could produce immunological toxicity. Any therapeutic strategy based on this pathway would therefore need to distinguish microglial CD28 biology from canonical T-cell CD28 biology, ideally through CNS-targeted delivery, microglia-selective targeting, or downstream pathway modulation rather than systemic CD28 agonism or blockade [20,48].

Another implication is that suppressive microglia may be most useful when combined conceptually with amyloid- or tau-directed therapies. Anti-amyloid antibodies can reduce amyloid burden, but clinical benefit has remained modest and safety concerns such as amyloid-related imaging abnormalities have limited enthusiasm in some health systems. A 2026 Cochrane review described debate over whether the overall clinical effects of anti-amyloid drugs are small or “trivial,” while also noting criticism that older and newer agents may have been pooled in ways that obscure differences among therapies [54]. Regardless of one’s interpretation, the debate reinforces a broader point: amyloid removal alone is unlikely to be the complete therapeutic answer for Alzheimer’s disease. Microglial state modulation could become relevant as an adjunct strategy that improves the brain’s inflammatory and clearance response to amyloid- or tau-directed interventions.

A suppressive-microglia framework also has implications for timing. If PU.1-low CD28-positive microglia arise in response to amyloid plaques, then they may be most relevant during a phase when amyloid pathology is established, but neuroinflammatory escalation and synaptic injury remain modifiable. Treating too early might be ineffective if the plaque niche required to induce the state is absent. Treating too late might be insufficient if neuronal loss, tau spread, and glial dysfunction have become self-sustaining. This argues for integrating microglial-state biomarkers into therapeutic development. Cerebrospinal fluid or blood biomarkers, PET imaging, and single-cell or spatial transcriptomic analyses of human tissue could help determine whether lymphoid-like suppressive microglial programs correlate with disease stage, plaque burden, tau pathology, inflammatory markers, or cognitive decline.

There is also a biomarker implication. If this microglial state proves relevant in humans, markers of the PU.1-low CD28-positive program could help identify patients whose disease biology is dominated by insufficient immune regulation rather than simply excess inflammation. This would be conceptually different from using generic inflammatory markers. A useful biomarker would need to report the presence, absence, or functional competence of a protective microglial state. Such a marker could be used to stratify patients, monitor response to microglia-modulating therapy, or explain why some individuals tolerate substantial amyloid pathology better than others.

The main therapeutic risk is that promoting suppressive microglia could accidentally suppress necessary immune functions. Microglia need to respond to injury, infection, debris, amyloid, tau, and vascular stress. An overly suppressive state might reduce harmful inflammation but also impair phagocytosis, antigen presentation, plaque containment, or tissue repair. This concern is especially relevant in older patients, who may already have immunosenescence, vascular disease, mixed pathologies, and reduced resilience. A successful therapy would therefore need to show not only reduced inflammatory markers, but preserved or improved clearance, synaptic integrity, neuronal survival, and cognition.

Overall, the therapeutic value of reviewing PU.1-low CD28-positive microglia is that it provides a more refined treatment hypothesis for Alzheimer’s disease. Instead of asking whether microglia should be activated or inhibited, the field can ask which microglial states should be encouraged, which should be restrained, and when each intervention should occur. The PU.1/CD28 axis suggests that Alzheimer’s plaques may induce not only damaging inflammatory responses but also endogenous attempts at immune regulation. If these suppressive microglia can be validated in human disease and safely manipulated, they could become part of a new class of therapies aimed at restoring balanced neuroimmune control rather than simply removing amyloid or suppressing inflammation.

## 6. Open Questions

The proposal that PU.1-low, CD28-positive microglia form a suppressive or neuroprotective state in Alzheimer’s disease raises several important questions that remain unresolved. The first and most basic question is whether this population exists in human Alzheimer’s disease in the same form described in experimental models. Ayata et al. reported that PU.1-low CD28-expressing microglia can reduce neuroinflammation and amyloid pathology, but a central task now is to determine whether comparable cells are present in human postmortem tissue, whether they are detectable in single-nucleus or spatial transcriptomic datasets, and whether their abundance correlates with plaque burden, inflammatory tone, synaptic preservation, or cognitive resilience [20]. This is especially important because human microglial states often overlap only partially with mouse-defined categories, and large human studies have shown that microglial populations in Alzheimer’s disease shift dynamically across disease contexts rather than fitting neatly into a few fixed states [22].

A second question concerns disease stage. Alzheimer’s disease develops over many years, with amyloid deposition generally preceding widespread tau pathology, neurodegeneration, and clinical dementia. If PU.1-low CD28-positive microglia are induced by plaque contact, then they may be most relevant during an amyloid-rich phase of disease, when microglia are still capable of mounting a protective plaque-associated response. However, it is not yet clear whether these cells persist, expand, disappear, or become dysfunctional as tau pathology and neurodegeneration progress. This matters therapeutically because microglial modulation may be beneficial at one stage but harmful at another. The TREM2 field illustrates this problem well: TREM2-related microglial activation may support amyloid plaque engagement, but its role in tau-dominant or later-stage disease is more controversial, and some authors have warned that timing may determine whether a microglia-targeted intervention behaves as a “friend” or a “foe” [55,56].

A third unresolved issue is whether lymphoid-like suppressive microglia are specific to amyloid pathology or whether they also occur in response to tau, vascular injury, α-synuclein pathology, or mixed dementia. The Ayata study is most directly linked to amyloid plaque-associated microglial biology, but human Alzheimer’s disease is rarely a pure amyloid disorder. It includes tau tangles, synapse loss, vascular pathology, astrocyte activation, oligodendrocyte stress, and age-related immune remodeling. Recent spatial and single-nucleus studies have emphasized that different pathological features may generate distinct glial niches, including plaque-associated microglial and astrocytic responses [57,58]. Therefore, one open question is whether PU.1-low CD28-positive microglia represent a plaque-specific regulatory state, a broader response to neurodegenerative injury, or one member of a larger family of suppressive microglial programs.

A fourth question concerns spatial organization. If these microglia are rare and plaque-proximal, bulk tissue methods may dilute their signal, and even single-nucleus sequencing may miss key features if the population is small or if relevant receptor proteins are poorly captured at the RNA level. Spatial transcriptomics, multiplexed immunohistochemistry, spatial proteomics, and high-resolution imaging will therefore be essential. Human spatial studies have already begun to identify plaque-glia niches and altered co-expression patterns around amyloid deposits, suggesting that the immediate plaque microenvironment is molecularly distinct from surrounding tissue [59]. The next step is to ask whether CD28-positive PU.1-low microglia occupy a reproducible position within these niches, whether they contact plaques directly, whether they interact with astrocytes or T cells, and whether their presence marks plaques that are less inflammatory or less neuritically damaging.

A fifth unresolved question is mechanistic. CD28 is classically understood as a T-cell co-stimulatory receptor, so its role in microglia cannot be assumed from lymphocyte biology. It remains unclear which ligands activate CD28 on microglia, whether canonical CD80/CD86 interactions are involved, whether neighboring microglia or infiltrating immune cells provide the relevant ligands, and whether CD28 signaling in microglia uses the same intracellular pathways as in T cells. It is also possible that CD28 expression is a marker of a broader PU.1-low regulatory program rather than the sole functional driver of suppression. The Ayata study points to CD28 and other lymphoid co-stimulatory or co-inhibitory receptor proteins as possible regulators of microglial responses, but the hierarchy of these molecules remains to be defined.

A sixth question is how PU.1 dosage should be interpreted. Human genetic evidence and the PU.1-low suppressive-microglia model suggest that reduced SPI1/PU.1 activity may be protective in some contexts. However, other studies indicate that PU.1 is essential for microglial identity and that reducing Spi1 can worsen Alzheimer’s-related pathology in certain models. A 2024 study reported that Spi1 knockdown aggravated amyloid aggregation, plaque accumulation, and gliosis, which argues strongly against a simple therapeutic model in which PU.1 is globally inhibited [36]. The open question is therefore not whether PU.1 should be increased or decreased overall, but how local, partial, and state-specific changes in PU.1 reshape microglial function. A useful review should emphasize this distinction because it separates the biological insight—PU.1 dosage helps govern microglial state—from the much riskier therapeutic idea of directly targeting PU.1.

A seventh question concerns the relationship between suppressive microglia and other Alzheimer ’s-associated microglial states. Do PU.1-low CD28-positive cells arise from disease-associated microglia, represent a branch of plaque-associated microglia, or form a distinct state trajectory? Are they related to antigen-presenting microglia, interferon-responsive microglia, lipid-associated microglia, or TREM2-dependent states? Large human microglial atlases and state-dynamics studies are beginning to show that microglia in Alzheimer’s disease occupy multiple transcriptional trajectories rather than discrete and permanent categories [22,47]. This raises the possibility that suppressive microglia are not a separate endpoint, but a transient branch that emerges only under specific combinations of plaque exposure, PU.1 reduction, ligand availability, and local inflammatory pressure.

An eighth question is whether these cells are truly protective or merely associated with protection. Functional studies in mice support a protective interpretation, but human validation will require more than detecting CD28 and low PU.1 expression. Researchers will need to test whether the presence of this state predicts reduced inflammatory pathology, preserved synapses, lower neuritic dystrophy, slower tau spread, or better cognitive outcomes after adjusting for amyloid load and disease stage. This distinction matters because many microglial states appear protective in one experimental context and harmful in another. The broader TREM2 literature again provides a cautionary example: pathways that support amyloid plaque response may have different consequences in tau-rich or late-stage disease [55,60].

A ninth open question is how these microglia interact with other cells in the Alzheimer’s brain. Microglia do not act alone; they exchange signals with astrocytes, neurons, oligodendrocytes, endothelial cells, border-associated macrophages, and sometimes peripheral immune cells. Spatial transcriptomic studies of plaque niches have highlighted microglia–astrocyte crosstalk around amyloid deposits, while newer single-nucleus studies across dementia subtypes suggest that microglia may also interact with oligodendrocyte-lineage cells in disease-specific ways [58,61]. A suppressive microglial state might therefore work not only by reducing microglial inflammation intrinsically, but also by changing astrocyte reactivity, complement activation, synaptic engulfment, vascular inflammation, or recruitment of peripheral immune cells.

A final and clinically important question is how this biology could be safely manipulated. If PU.1-low CD28-positive microglia are protective, then one therapeutic goal might be to expand or stabilize them. Yet this goal is complicated by the central role of CD28 in peripheral T-cell biology and the essential role of PU.1 in myeloid identity. Any intervention that broadly stimulates or inhibits CD28 signaling could affect systemic immune function, while any intervention that globally lowers PU.1 could impair microglial viability, phagocytosis, or host defense. The safer strategy may be to identify downstream effectors of the suppressive program, define plaque-niche signals that naturally induce it, and develop CNS- or microglia-targeted approaches that preserve beneficial plaque containment while limiting inflammatory damage.

## 7. Conclusions

The emerging concept of PU.1-low, CD28-positive lymphoid-like microglia provides a focused framework for rethinking neuroimmune regulation in Alzheimer’s disease. Rather than viewing microglia only as plaque-clearing or pro-inflammatory cells, this model suggests that the Alzheimer’s brain may also contain specialized microglial programs that actively restrain inflammation within the plaque niche. The value of this review lies in integrating SPI1/PU.1 genetics, microglial transcriptional regulation, plaque-associated microglial states, CD28/B7-family signaling, and therapeutic microglial reprogramming into a single state-based perspective.

A central conclusion is that PU.1/CD28 biology should be interpreted through the lens of dosage, timing, and cellular context. Lower SPI1/PU.1 activity may be protective in some settings, but PU.1 remains essential for microglial identity and function. Therefore, PU.1 should not be viewed as a simple target for global inhibition. Similarly, CD28 expression in microglia should not be directly extrapolated from classical T-cell biology. If PU.1-low CD28-positive microglia are protective, future strategies will likely need to modulate downstream regulatory programs or plaque-niche signals rather than broadly manipulating PU.1 or CD28.

At present, lymphoid-like suppressive microglia remain a promising but still incompletely validated concept. Key questions include whether these cells are reproducibly present in human Alzheimer’s disease, whether they are restricted to amyloid-rich plaque niches or also occur in tau and mixed pathologies, and whether their abundance predicts reduced neuroinflammation, preserved synapses, or slower clinical progression. Addressing these questions through spatial transcriptomics, single-cell multiomics, proteomics, and human postmortem validation will determine whether this state represents a rare experimental observation or an important protective mechanism in Alzheimer’s disease.

## Figures and Tables

**Table 1:** Key studies informing the proposed PU.1/CD28 regulatory-axis framework in Alzheimer’s disease microglia.

Relevance to the Present Review	Main Finding	Experimental or Analytical Approach	Study
Established microglia as genetically embedded contributors to Alzheimer’s disease rather than secondary inflammatory bystanders.	Rare coding variants in *PLCG2*, *ABI3*, and *TREM2* implicated microglia-mediated innate immunity in Alzheimer’s disease.	Human genetic study of Alzheimer’s disease risk variants	Sims et al. [15]
Supports the idea that microglial disease states are actively regulated transcriptional programs.	Identified a TREM2–APOE-regulated microglial neurodegenerative phenotype characterized by loss of homeostatic genes and induction of lipid-handling, phagocytic, and inflammatory programs.	Transcriptomic and mechanistic analysis of microglia in neurodegenerative disease models	Krasemann et al. [16]
Provides a foundational framework for interpreting Alzheimer ’s-associated microglia as heterogeneous, disease-linked states.	Described disease-associated microglia (DAM), a microglial state enriched around pathology and characterized by staged activation involving TREM2-dependent and TREM2-independent components.	Single-cell RNA sequencing in mouse models of neurodegeneration	Keren-Shaul et al. [17]
Demonstrates that microglial heterogeneity is relevant to human Alzheimer’s disease, while also highlighting differences between mouse and human states.	Identified human microglial subsets associated with Alzheimer’s disease and disease-related gene-expression signatures.	Single-cell RNA sequencing of human microglia	Olah et al. [18]
Supports the view that Alzheimer ’s-associated microglial responses are diverse and not reducible to one universal disease state.	Revealed TREM2-dependent and TREM2-independent cellular responses in Alzheimer’s disease across human and mouse tissue.	Human and mouse single-nucleus transcriptomics	Zhou et al. [21]
Provides a basis for considering PU.1-low CD28-positive microglia as a possible rare, transient, or spatially restricted state within broader microglial trajectories.	Showed that human microglia shift along disease-associated state trajectories during Alzheimer’s disease progression.	Large-scale human microglial transcriptomic analysis across Alzheimer’s disease progression	Sun et al. [22]
Illustrates a harmful microglial mechanism and emphasizes why protective versus damaging microglial functions must be distinguished.	Demonstrated that complement and microglia mediate early synapse loss in Alzheimer’s mouse models.	Alzheimer’s disease mouse models with complement-pathway analysis	Hong et al. [23]
Shows that microglial effects occur within a broader glial network involving astrocytes, complement, synapses, amyloid, and tau pathology.	Reported C1q-dependent excitatory and inhibitory synapse elimination involving astrocytes and microglia.	Alzheimer’s disease mouse models combining amyloid and tau-related pathology	Dejanovic et al. [24]
Supports the broader principle that transcription-factor dosage can redirect microglial state, relevant to interpreting PU.1-low CD28-positive microglia.	Found that partial or total MEF2C reduction induced disease- and lipid-associated macrophage/microglial programs linked to lysosomal, phagocytic, lipid-handling, and cholesterol-efflux pathways.	Human iPSC-derived microglia/macrophage models and human triculture Alzheimer’s disease model	Podlesny-Drabiniok et al. [25]
Places PU.1 within a broader enhancer-regulatory network that controls microglial identity and disease-state transitions.	Showed that IRF8 helps configure the postnatal microglial enhancer landscape together with SALL1 and PU.1, and that IRF8 disruption can induce disease-associated and interferon-related microglial programs.	Microglial enhancer and transcription-factor analysis involving IRF8, SALL1, and PU.1	Saeki et al. [28]
Provides mechanistic support for the idea that SPI1/PU.1 dosage influences microglial phenotype and inflammatory tone.	Showed that Alzheimer’s-associated PU.1 expression levels regulate microglial inflammatory responses.	Experimental modulation of Alzheimer’s-associated PU.1 expression levels in microglial models	Pimenova et al. [32]
Provides an important caution that PU.1 cannot be viewed simply as a target for broad inhibition.	Demonstrated that PU.1 is critical for microglial viability and function.	Human brain microglia analysis	Smith et al. [33]
Reinforces the concept that PU.1 effects are dosage-sensitive rather than purely protective or harmful.	Showed that modest changes in *Spi1* dosage can alter microglial function in ways relevant to Alzheimer’s disease.	Spi1 dosage study in mice	Jones et al. [34]
Strongly supports a cautious interpretation of PU.1 modulation and argues against simple global PU.1 inhibition.	Examined how SPI1-mediated transcriptome remodeling affects Alzheimer ’s-related phenotypes and showed that reducing Spi1 can worsen some pathological features.	SPI1 knockdown and overexpression in Alzheimer’s disease-related mouse models	Kim et al. [37]
Central study for the present review; provides the basis for the proposed lymphoid-like suppressive microglial framework.	Identified PU.1-low CD28-positive microglia with lymphoid-like gene expression and proposed that these cells may suppress neuroinflammation and reduce amyloid pathology.	Experimental and transcriptomic analysis of plaque-associated microglia	Ayata et al. [20]
Supports the relevance of lymphoid-like and adaptive immune signaling in Alzheimer’s disease neuroimmune regulation.	Reported altered T-cell reactivity in early Alzheimer’s disease, including adaptive immune changes across disease stages.	Human immune profiling in early Alzheimer’s disease	Rickenbach et al. [37]
Demonstrates that PU.1-associated inflammatory programs may be pharmacologically modulated, supporting the therapeutic relevance of state-specific microglial reprogramming.	Identified A11, a compound that recruits HDAC/MECP2-containing repressive complexes to PU.1 motifs and reduces neuroinflammation and neuropathology in preclinical models.	Small-molecule modulation of PU.1-regulated inflammatory transcription in human iPSC-derived microglia-like cells and mouse models	Ralvenius et al. [38]

## Data Availability

No new data were created or analyzed in this study. Data sharing is not applicable to this article.
